# Antiproliferative effect of growth hormone-releasing hormone (GHRH) antagonist on ovarian cancer cells through the EGFR-Akt pathway

**DOI:** 10.1186/1477-7827-8-54

**Published:** 2010-05-28

**Authors:** Jian Guo, Andrew V Schally, Marta Zarandi, Jozsef Varga, Peter CK Leung

**Affiliations:** 1 Department of Obstetrics & Gynaecology, Child and Family Research Institute, UBC, Vancouver, Canada; 2 School of Preclinical Medicine, Beijing University of Chinese Medicine, Beijing, China; 3 Veterans Affairs Medical Center and Departments of Pathology and Medicine, Division of Hematology/Oncology, University of Miami Miller School of Medicine, Miami, FL 33125, USA

## Abstract

**Background:**

Antagonists of growth hormone-releasing hormone (GHRH) are being developed for the treatment of various human cancers.

**Methods:**

MTT assay was used to test the proliferation of SKOV3 and CaOV3. The splice variant expression of GHRH receptors was examined by RT-PCR. The expression of protein in signal pathway was examined by Western blotting. siRNA was used to block the effect of EGFR.

**Results:**

In this study, we investigated the effects of a new GHRH antagonist JMR-132, in ovarian cancer cell lines SKOV3 and CaOV3 expressing splice variant (SV)1 of GHRH receptors. MTT assay showed that JMR-132 had strong antiproliferative effects on SKOV3 and CaOV3 cells in both a time-dependent and dose-dependent fashion. JMR-132 also induced the activation and increased cleaved caspase3 in a time- and dose-dependent manner in both cell lines. In addition, JMR-132 treatments decreased significantly the epidermal growth factor receptor (EGFR) level and the phosphorylation of Akt (p-Akt), suggesting that JMR-132 inhibits the EGFR-Akt pathway in ovarian cancer cells. More importantly, treatment of SKOV3 and CaOV3 cells with 100 nM JMR-132 attenuated proliferation and the antiapoptotic effect induced by EGF in both cell lines. After the knockdown of the expression of EGFR by siRNA, the antiproliferative effect of JMR-132 was abolished in SKOV3 and CaOV3 cells.

**Conclusions:**

The present study demonstrates that the inhibitory effect of the GHRH antagonist JMR-132 on proliferation is due, in part, to an interference with the EGFR-Akt pathway in ovarian cancer cells.

## Background

Ovarian cancer is the second most common gynecologic cancer among women and ranks as the most common cause of death from gynecologic malignancies in the western world [1]. Ovarian cancer is difficult to diagnose at an early stage and most patients are discovered at advanced stage due to lack of effective early screening methods [2]. Despite the use of cytoreductive surgery and systemic chemotherapy, the metastatic disease remains generally incurable with a 5-year survival rate of around 40% for these patients [1]. Therefore, it is critical to introduce more effective therapeutic agents for the management of this malignancy.

Antagonists of growth hormone-releasing hormone (GHRH) are being developed for the treatment of various cancers [3,4]. Since 1994, many antagonistic analogs of GHRH have been synthesized in the laboratories of one of us [3]. GHRH antagonists were shown to inhibit the proliferation both in vivo and in vitro of various human cancers, including pancreatic [5], colorectal [6], prostatic [7-10], breast [11,12], renal [13], glioblastomas [14], osteosarcomas and Ewing sarcomas [15,16], lung carcinomas [17,18], lymphomas [19], as well as ovarian [20] and endometrial cancer [21]. GHRH antagonists can suppress tumor growth by indirect and direct pathways. The indirect action is mediated through the suppression of production of the pituitary GH and hepatic insulin-like growth factor I (IGF-I), which results in growth inhibition of some tumors [3,22,23]. However, much evidence from both in vivo and in vitro experiments shows that GHRH antagonists can also directly suppress tumor cells growth. Thus, the growth of various human cancers was suppressed without any involvement of the hypothalamic GHRH/pituitary GH/hepatic IGF-I axis [3]. The effect occurs through the disruption of the autocrine/paracrine production of IGF-I and/or IGF-II in tumors [3,24-26] by GHRH antagonists, or through the blockade of the stimulatory loop formed by tumoral GHRH and its receptors in tumors [3,27-34].

Four splice variants (SVs) of GHRH receptors (GHRHR) have been demonstrated in various human cancers and cancer cell lines [3,27]. One of the four isoforms, SV1 has the greatest structural similarity to the pituitary GHRHR and is probably the main SV that mediates the effects of GHRH and its antagonists in tumors [3,27-34].

JMR-132 is a novel, highly potent GHRH antagonist. JMR-132 has been shown to inhibit human breast cancer [35,36], prostate cancer [37] and lung cancer [38,39], but the effect on ovarian cancer cells has not been reported so far. Knowledge about the mechanisms of GHRH antagonists involved in the antiproliferative effects, including apoptosis and cell circle arrest, is limited. Some recent studies have shown that cAMP [40], PKC [41], p21 [39] and p53 [42,43] may participate in mediating the effect of GHRH antagonists on inhibition of proliferation and the induction of apoptosis.

It has been also proposed that EGFR plays an important role in ovarian cancer, since this receptor is overexpressed in nearly 75% of primary ovarian cancers [44]. The over-expression of EGFR might be related to advanced-stage disease and poor prognosis [45]. EGFR regulates essential cellular functions, including proliferation, apoptosis, migration, and differentiation. Various ligands, such as EGF, amphiregulin (AR) and transforming growth factor-α (TGFα), are known to bind to EGFR, and will stimulate receptor homodimerization or heterodimerization for initiation of signal transduction. Published data demonstrates that the effects of EGFR signaling on cell proliferation and survival are mediated by PI3K-Akt pathways. Activation of EGFR results in the activation of the lipid kinase, PI3K, generating the second messenger phosphatidylinositol 3,4,5-trisphosphate(PIP3), which in turn activates Akt [46]. Thus, EGFR signaling has become an important target in anticancer drug development due to its ability to suppress apoptosis and to control tumor cell proliferation and migration [47,48]. Previous in vivo studies in lung cancer showed that decreased EGFR level might be involved in the antiproliferative effect of GHRH antagonists [49,50], but it hasn't been reported in ovarian cancer cell lines by JMR-132 treatment.

In this study, we for the first time demonstrate that the antiproliferative effect of JMR-132 in SKOV3 and CaOV3 cells occurs through EGFR pathway-dependent down-regulation of the p-Akt level, and consequently leads to the induction of cleaved caspase3, which indicate that SKOV3 and CaOV3 cells have experienced apoptosis. Our work also suggests that JMR-132 could be useful in the treatment of ovarian cancers.

## Methods

### Reagents

The GHRH antagonist JMR-132 was synthesized in the laboratories of one of us. Its structure is [PhAc^0^-Tyr^1^, D-Arg^2^, Phe(4-Cl)^6^, Ala^8^, Har^9^, Tyr(Me)^10^, His^11^, Abu^15^, His^20^, Nle^27^, D-Arg^28^, Har^29^] hGH-RH(1-29)NH2, where Abu is α-aminobutyric acid, Har is homoarginine, Nle is norleucine, PhAc is phenylacetyl and Tyr(Me) is o-methyltyrosine, as reported previously [3]. The PI3K inhibitor LY294002 and EGF were purchased from Sigma (St. Louis, MO, USA).

### Cell culture

Human epithelial ovarian cancer cell lines SKOV3 and CaOV3 classified as adenocarcinomas were obtained from the American Type Culture Collection (ATCC, Moanassas, USA). The cells were cultured in Dulbecco's minimum essential medium (DMEM) (Invitrogen Inc., Burlington, ON, Canada) supplemented with 10% fetal bovine serum (FBS) (HyClone Laboratories Inc., Logan, UT), 100 U/ml penicillin and 100 mg/ml streptomycin (Life Technologies, Inc., Rockville, MD, USA) and incubated at 37°C in a humidified incubator with 5% CO_2_. The cells were grown to 80% confluence and incubated with serum free medium overnight before treatment with JMR-132, EGF and PI3K inhibitor LY294002.

### MTT assay

Cell viability was estimated by the [3-(4,5-dimethylthiazol-2-yl)-2,5-diphenyltetrazoliumbromide] (MTT) (Sigma-Aldrich Corp.) assay. CaOV3 or SKOV3 cells were seeded into 96-well dishes (SKOV3, 2 × 10^3 ^cells/well; CaOV3, 3 × 10^3 ^cells/well) with DMEM containing 10% FBS. DMEM medium (180 ul) was added to each well. After 24 hours of incubation, the cells were treated with GHRH antagonist JMR-132 (100 nM), EGF (10 ng/ml) and PI3K inhibitor LY294002 (10 μM). The MTT colorimetric assay was performed to detect tumor cell viability after 24 h, 48 h, 72 h and 96 h of incubation. The cells were then incubated at 37°C with 20 μl of MTT solution (5 mg/ml in PBS) for 4 h. The supernatants were removed and the cells were solubilized in DMSO (200 μl) for 15 min. The OD at 490 nm was determined using an ELISA reader (Fisher Scientific Ltd., Ottawa, Canada).

### mRNA isolation and RT-PCR

After treatment with JMR-132, the medium was removed from each culture dish and RNA was extracted using Trizol (Invitrogen). Total RNA (2 μg) was reverse-transcribed into first-strand cDNA (GEHealthcare Bio-Science, Piscataway NJ, USA) following the manufacturer's protocol. The primer for the analysis of mRNA expression of GHRH receptor SV1 has been described [28,29]. The primers used were 5'-CCT ACT GCC CTT AGG ATG CTG G-3' (sense) and 5'-GCA GTA GAG GAT GGC AAC AAT G-3' (antisense). The PCR conditions were 1 cycle at 95°C for 3 min, followed by 40 cycles at 95°C for 30 s, 60°C for 30 s and 72°C for 60 s. Four microliters of the PCR product were then used to perform a second PCR. The primers used were 5'-GCA CCT TTG AAG CCA GAG AAG G-3' (sense) and 5'-CAC GTG CCA GTG AAG AGC ACG G-3' (antisense). The product length was 720 bp. The primers for human GAPDH were 5'-ATGTTCGTCATGGGTGTGAACCA-3' (sense) and 5'-TGGCAGGTTTTTCTAGACGGCAG-3' (antisense). The cycling conditions were 94°C for 5 m, followed by 25 cycles of 94°C for 30 s, 55°C for 30 s and 72°C for 60 s. The product length was 373 bp. The PCR products were electrophoresed on a 2% agarose gel, stained with ethidium bromide and visualized under ultraviolet light.

### Western blotting

The cells were homogenized in RIPA lysis buffer containing 50 nM Tris-HCl pH 7.4, 150 mM NaCl, 1% Nonidet P-40 and 0.1% SDS, supplemented with protease inhibitors (PMSF) and phosphatase inhibitors (1 mM NaF). After centrifugation (12000 × g, 15 min), the supernatants were collected and the protein concentrations were determined by spectrophotometer. After boiling at 98°C for 5 min, 50 μg of total protein per lane was added. The proteins were separated by 8-10% SDS-PAGE and transferred to a nitrocellulose membrane. The nitrocellulose sheet was incubated in 5% nonfat milk for 1 h at room temperature and then exposed to the primary antibodies against caspase3, p-Akt, Akt, EGFR and β-actin (1:1000) at 4°C overnight. After three washes in TBS, the membranes were incubated with the correct peroxidase-conjugated secondary antibodies for 1 h at room temperature and washed again with TBS. The bands were visualized with Supersignal West Pico Chemiluminescent Substrate (Pierce Co., USA).

### In vitro transfection with small interfering RNAs (siRNAs)

siRNAs targeting EGFR were synthesized by Invitrogen (Burlington, ON, Canada). In addition, a nonspecific scrambled siRNA was purchased from Invitrogen and used as a control. The siRNA transfection was performed according to the manufacturer's instructions (Invitrogen). Briefly, 24 hours before transfection, 6-well plates were seeded with 1 × 10^4 ^cells per well in 2 ml of culture medium. The cells were transfected with EGFR (100 nM final concentration) or scrambled siRNA with 1 ml of lipofectamine iMAX reagent according to the manufacturer's protocol. 48 hours later, the cells were treated with JMR-132. For the MTT assay, the cells from the 6-well plate were re-seeded in a 96-well plate 48 hours after transfection.

### Statistical analysis

Data were subjected to one-way ANOVA and differences were determined by Tukey's multiple comparison test. Each experiment was repeated three times. Data are shown as the means of three individual experiments and presented as the mean ± SEM. *P *< 0.0 5 was considered statistically significant.

## Results

### The expression of mRNA for GHRHR SV1 in SKOV3 and CaOV3 cells

**Figure 1:**
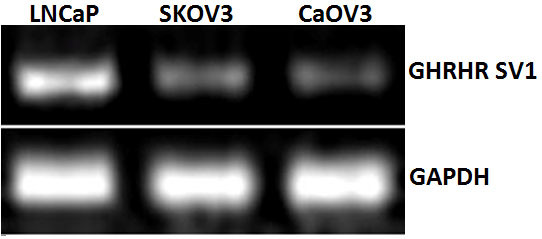
**The expression of mRNA for GHRHR SV1 in SKOV3 and CaOV3 human ovarian cancer cells**. The prostate cancer cell line LNCaP was used as a positive control. Human GAPDH was used as an internal control.

Previous studies showed that GHRHR SV1 was expressed in different cancer cells. As shown in Figure 1, mRNA for GHRHR SV1 was expressed in both SKOV3 and CaOV3 cancer cells. LNCaP cells are androgen-sensitive human prostate adenocarcinoma cells. It has been reported that splice variants 1(SV1) of GHRH receptors was expressed in LNCaP cells [27-29]. However, the level of GHRHR SV1 mRNA in SKOV3 and CaOV3 cells is lower than that in LNCaP prostate cancer cells, which were used as a positive control.

### The antiproliferative effect of JMR-132 on the SKOV3 and CaOV3 cell lines

**Figure 2:**
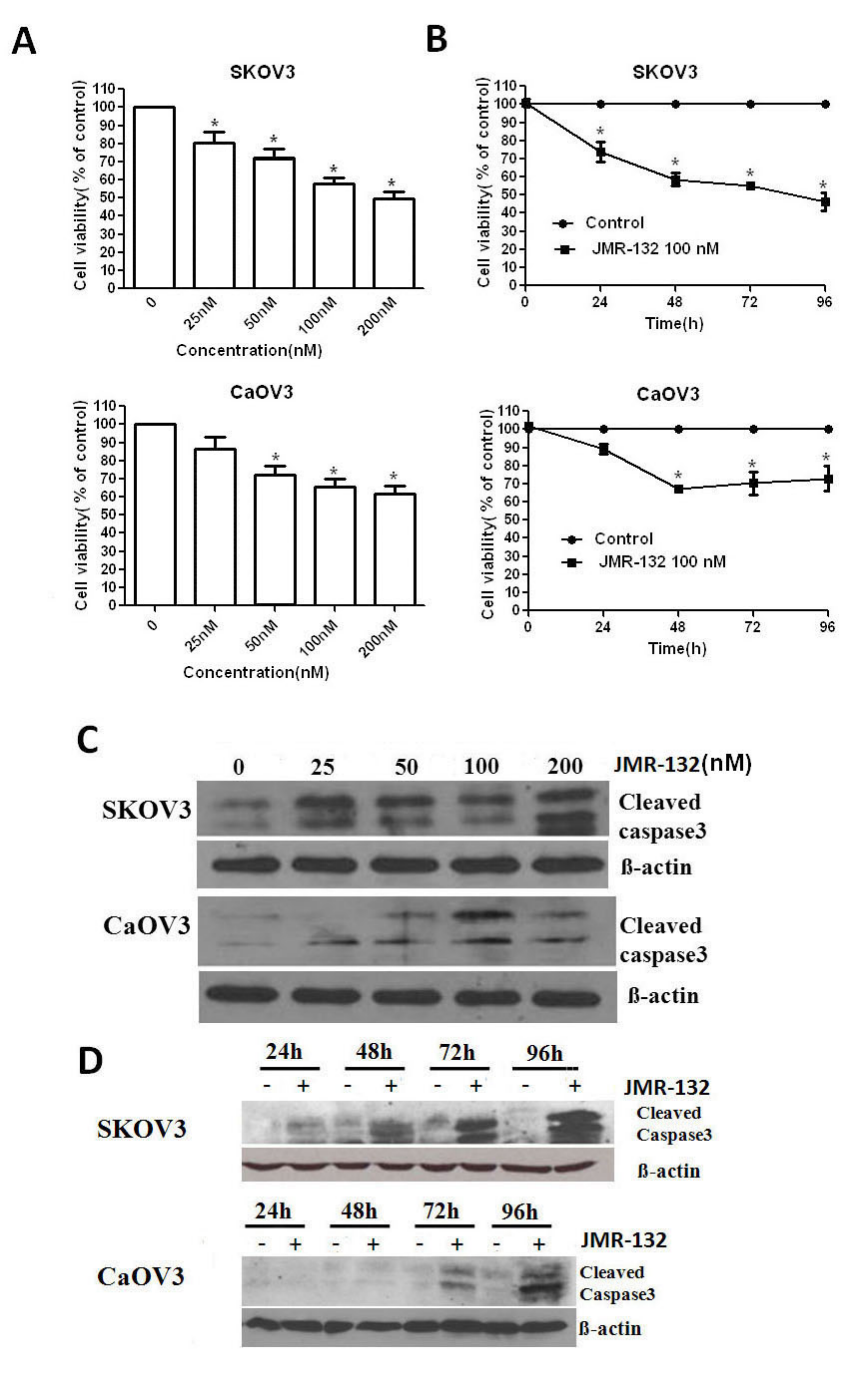
**Antiproliferative effect of JMR-132 on SKOV3 and CAOV3 cells**. SKOV3 (2000/well) and CaOV3 (3000/well) were treated with JMR-132 at a different dose (25, 50, 100 and 200 nM) per day for 2 days (A), (C) and with 100 nM JMR-132 per day for different periods of time (24, 48, 72 and 96 h) (B) (D). MTT assay showed that proliferation was inhibited in a dose (A) and time (B) dependent manner after JMR-132 treatment. * P < 0.05 compared to the no treatment group. The expression of cleaved caspase3 increased in a dose (C) and time (D) dependent manner. The results indicated that JMR-132 treatment induced apoptosis in these cells.

The growth of the SKOV3 and CaOV3 cells was checked by the MTT assay. As shown in Figure 2A and 2B, both the treatment with a different dose per day of JMR-132 for 2 days and treatment with 100 nM JMR-132 per day for 1 to 4 days resulted in a significant decrease in the growth of SKOV3 and CaOV3 cells compared to the control group. However, the effect in SKOV3 cells was stronger than that in CaOV3 cells. The maximum inhibition occurred after continuous treatment for 4 days with 100 nM JMR-132 and reached about 60% in SKOV3 cells. These data showed that JMR-132 can suppress growth of SKOV3 and CaOV3 cells in a dose- and time-dependent manner.

To further clarify whether the antiproliferative effect is due to growth inhibition or apoptosis induced in the SKOV3 and CaOV3 cells, we checked the expression of cleaved caspase3 which is a known marker of apoptosis. As shown in Figure 2C and 2D, after treatment with JMR-132, the level of cleaved caspase3 obviously increased in time- and dose-dependent manner. As seen from growth inhibition in SKOV3 and CaOV3 cells, the increase in the level of cleaved caspase3 in SKOV3 cells was greater than that in CaOV3 cells. Thus apoptosis might contribute to the antiproliferative effect induced by JMR-132 treatment in SKOV3 and CaOV3 cells.

### Decrease in EGFR level after JMR-132 treatment in SKOV3 and CaOV3 cells

**Figure 3:**
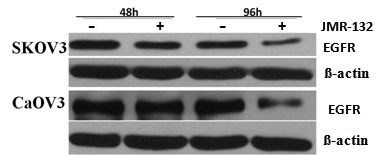
**The expression of EGFR in SKOV3 and CaOV3 cells decreased after JMR-132 treatment**. The protein level of EGFR decreased after treatment with 100 nM JMR-132 per day for 2 and 4 days. β-actin was used as an internal control. The data are from one experiment and are representative of the three separate experiments.

EGFR plays an important role in proliferation in cancer research. To determine whether the mechanism of the antiproliferative effect of JMR-132 is associated with EGFR, we studied the EGFR protein expression. As shown in Figure 3, EGFR levels decreased significantly in SKOV3 and CaOV3 cells after 48 to 96 hours of treatment with 100 nM JMR-132. Our data indicated therefore that EGFR might be involved in the regulation of the effect of JMR-132.

### JMR-132 attenuates the effect of EGF-induced p-Akt activation

**Figure 4:**
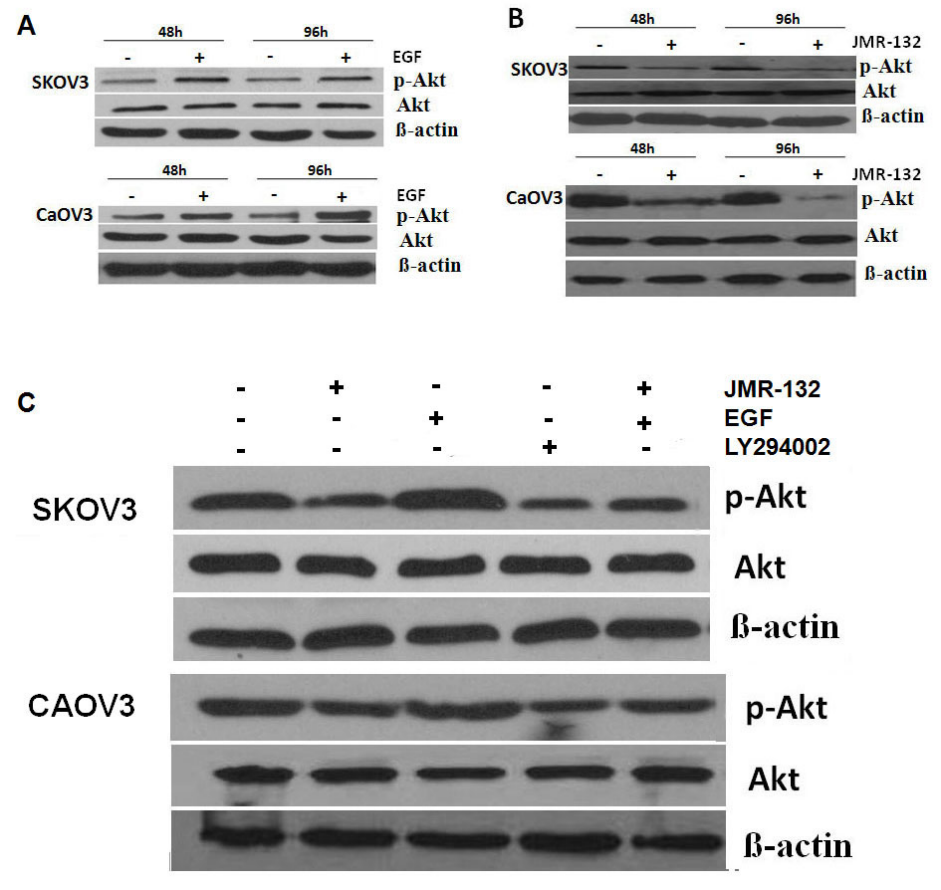
**JMR-132 attenuated the effect of EGF-induced p-Akt activation**. A. p-Akt was activated after EGF (10 ng/ul) treatment without changes in Akt. β-actin was used as an internal control. B. p-Akt expression decreased after treatment with 100 nM JMR-132 per day for 2 and 4 days without changes in Akt level. C. JMR-132 attenuated the effect of EGF-induced p-Akt expression. JMR-132 (100 nM) and PI3K specific inhibitor LY294002 (10 nM) were used separately to continuously treat SKOV3 and CaOV3 cells for 2 days. PI3K specific inhibitor LY294002 was used as a positive control. β-actin was used as an internal control. EGF was added 15 min prior to harvesting.

To characterize whether the antiproliferative effect is related to the decreased level of EGFR, we measured EGFR downstream signaling by Western blotting of Akt and apoptotic-associated caspase3. We first confirmed that EGF (10 ng/μl) induced p-Akt activation (Figure 4A). The treatment of SKOV3 and CaOV3 cells with JMR-132 led to a decrease in p-Akt (Figure 4B), which suggested that the antiproliferative effect might result from the PI3K-Akt pathway. Using the PI3K-specific inhibitor LY294002 as a positive control, we treated the cells with JMR-132 alone or in combination with EGF and found that JMR-132 still inhibited p-Akt expression as compared to the control group. In addition, JMR-132 attenuated the EGF-induced increase in the p-Akt level. Our results indicate that the EGFR-Akt pathway plays a role in regulating the function of the GHRH antagonist JMR-132.

### JMR-132 abolishes the pro-proliferative and anti-apoptotic effect of EGF

**Figure 5:**
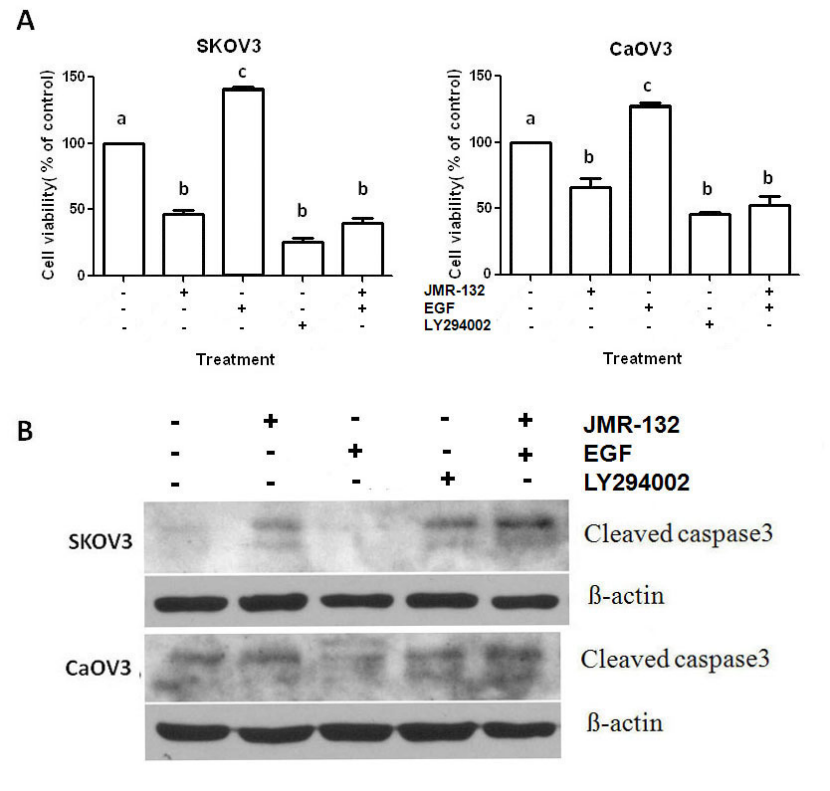
**JMR-132 abolished the pro-proliferative and anti-apoptotic effect of EGF**. A. Using the MTT assay, it was determined that the growth of SKOV3 and CaOV3 cells was significantly inhibited after continuous treatment with 100 nM JMR-132 for 2 days in combination with EGF (10 μg/ml). Letters (a, b, c) between pairs indicate significant differences (P < 0.05). B. The expression of cleaved caspase3 increased during treatment with 100 nM JMR-132 per day for 2 days in combination with EGF (10 μg/ml). β-actin was used as an internal control. EGF was added 15 min prior to harvesting in (A) and (B).

To continue the investigation of the EGFR-Akt pathway, we examined the proliferation of SKOV3 and CaOV3 cells after treatment with JMR-132 alone or in combination with EGF. As shown in Figure 5A, the growth of SKOV3 and CaOV3 cells was significantly inhibited after treatment with JMR-132 alone or in combination with EGF. The antiproliferative effect was stronger in SKOV3 cells than in CaOV3 cells. We also examined the expression of caspase3 in SKOV3 and CaOV3 cells after the same treatment. Similar to the proliferation data, JMR-132 upregulated the level of cleaved caspase3, which suggested that apoptosis occurred. However, EGFR plays an antiapoptotic role and no cleaved caspase3 was induced in the EGF treatment group. Interestingly, treatment with JMR-132 in combination with EGF again led to an increase in the level of the cleaved caspase3. The apoptosis data are consistent with the proliferation data, suggesting that the antiproliferative effect is mainly due to the cells undergoing apoptosis and partially to the antiproliferative effect of JMR-132 acting on the EGFR-Akt pathway.

### Lack of effect of treatment with JMR-132 after EGFR siRNA transfection in SKOV3 and CaOV3 cells

**Figure 6:**
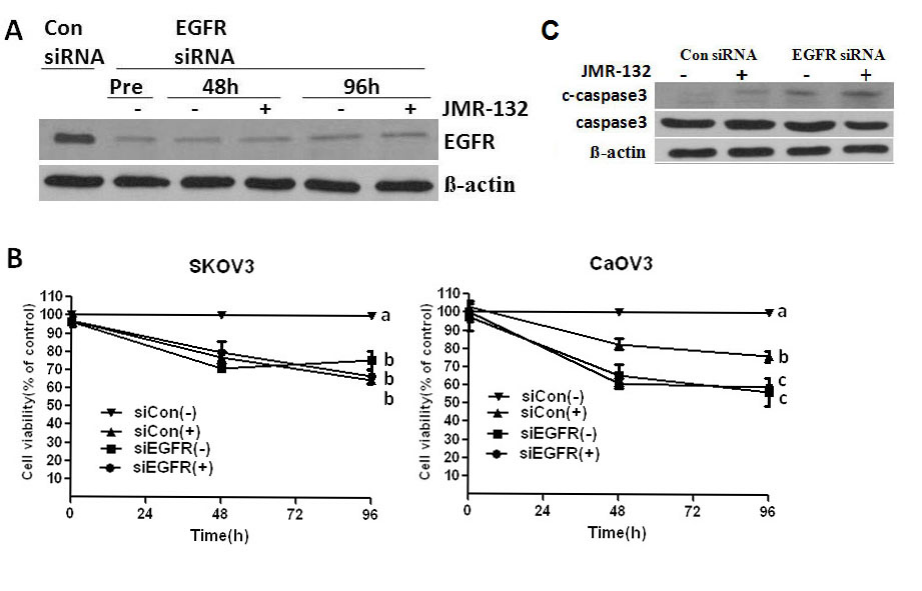
**No changes were detected after transfection with EGFR siRNA, either with or without JMR-132 treatment**. A. EGFR was knocked down after transfection with EGFR siRNA for various periods of time. After transfection with 100 nM EGFR siRNA for 48 hours, we reseeded the cells for the MTT assay. Pretreatment: after cell reseeding; 48 and 96 hours: treatment with 100 nM JMR-132 per day for 2 and 4 days after cell reseeding. The EGFR expression was still lower when compared to the cells transfected with 100 nM control siRNA. B. No changes were found by the MTT assay after transfection with EGFR siRNA, with or without JMR-132 treatment. The cells (SKOV3 and CaOV3: 4000/well) were reseeded in a 96-well plate after transfection with control siRNA or EGFR siRNA for 2 days. After 2 and 4 days of treatment with 100 nM JMR-132, the MTT assay was performed. Letters (a, b, c) indicate significant differences (P < 0.05) between pairs. C. No changes were detected in cleaved caspase3 expression after transfection with EGFR siRNA, either with or without JMR-132 treatment in SKOV3 cells. After transfection with 100 nM EGFR siRNA for 48 hours, the SKOV3 cells were treated with 100 nM JMR-132 per day for 2 days. β-actin was used as an internal control.

To further confirm that EGFR is involved in mediating the function of JMR-132, we knocked down EGFR using 100 nM EGFR siRNA. After transfection with 100 nM EGFR siRNA for 2 days, the EGFR expression was significantly decreased. Since we reseeded the cells in a 96-well plate after stable EGFR siRNA transfection to examine the cell proliferation with or without JMR-132 treatment for 2 and 4 days, we checked the long-term expression of EGFR, which was still low (Figure 6A). In the MTT assay (Figure 6B), we found that the growth of SKOV3 and CaOV3 cells was inhibited after EGFR was knocked down, which indicated that EGFR plays a major role in cell proliferation. More importantly, no changes in proliferation were detected after transfection with EGFR siRNA, following JMR-132 treatment. However, there was still an inhibitory effect of JMR-132 in the control siRNA transfection group. The same results were found for cleaved caspase3 expression in SKOV3 cells (Figure 6C). These data suggest that the antiproliferative effect of JMR-132 was reduced. Consistent with the results for proliferation, the level of cleaved caspase3 was slightly increased after knocking down EGFR, which indicates that a decrease in the EGFR level results in apoptosis. We then examined the expression of cleaved caspase3 with or without JMR-132 treatment after knocking down EGFR, and no obvious changes between the two groups were found. These data confirmed that the antiproliferative effect of JMR-132 is partially due to the decrease in EGFR. In addition, the effects on growth inhibition and apoptosis induction after JMR-132 treatment were greater in the EGFR siRNA group compared to the control siRNA group, which confirmed the importance of EGFR in cell proliferation.

## Discussion

Since 1994, various GHRH analogs with different structural features, including GHRH agonists and antagonists, have been synthesized [3]. Many studies were performed on the effects of the GHRH antagonists on different experimental cancers or human cancer cell lines. It was found that the treatment with the GHRH antagonists inhibit the growth of many human cancers, including ovarian cancer cell lines [3,20]. Although these observations have demonstrated the inhibitory role of GHRH antagonists on the proliferation of ovarian cancer [3,20], the effects of GHRH antagonists on other aspects of cancer phenomena, such as apoptosis, is poorly understood. It has been also shown that the GHRH antagonist JMR-132 had antiproliferative effects in lung, breast and prostate cancers [35-39], but the effect of JMR-132 on ovarian cancer cell lines was not reported. Thus, we conducted the present study to examine the effect of JMR-132 on two ovarian cancer cell lines, SKOV3 and CaOV3, and to investigate the mechanisms involved.

Previous studies showed that SV1 of GHRHR expressed in several cancers, may mediate the direct inhibitory effect of GHRH antagonists [3,27-34]. Here, we examined the expression of GHRHR SV1 in SKOV3 and CaOV3 cancer cell lines. Our data are in agreement with previous studies, indicating that GHRHR SV1 may play a functional role in regulating the effect of GHRH antagonists. The presence and the role of pituitary type of GHRH receptor [3,17] in ovarian cancer lines was not investigated in this study.

The effect of the GHRH antagonist JMR-132 on ovarian cancer cells was primarily studied with the MTT assay. Treatment with JMR-132 significantly inhibited the proliferation of SKOV3 and CaOV3 ovarian cancer cell lines. We then considered whether this antiproliferative effect was due to growth inhibition or apoptosis induction. Cleaved caspase3, the large fragment (17-19 kDa) of activated caspase3, results from pro-caspase3 (37KD) and is a known marker of apoptosis. It was observed that JMR-132 induced an increase in activated caspase3 in a time- and dose-dependent manner, which indicates that the cells were undergoing apoptosis. Both cell cycle arrest and apoptosis lead to growth inhibition. We also looked for changes in the cell cycle phase using flow cytometry; the G1 phase showed no obviously increase (data not shown). Results from this study demonstrate that growth suppression induced by JMR-132 treatment may be due to promoting apoptosis, not by cell cycle arrest. In short, JMR-132 could activate and increase cleaved caspase3 expression in ovarian cancer cells, which resulted in apoptosis, thus inhibiting the cell proliferation. Therefore, the apoptosis induced by JMR-132 treatment was a main contributor to proliferative inhibition.

The overexpression of EGFR results in an increased proliferation of solid tumors, including ovarian cancer [45]. Moreover, EGFR expression correlates with tumor resistance to chemotherapy [45] and indicates a poor prognosis. Previous studies suggested that the antiproliferative effect of the GHRH antagonist might involve EGFR [49,50]. However, the molecular mechanisms linking GHRH antagonists to the EGFR pathway in ovarian cancer cells were not well established. Our findings showed that the protein level of EGFR decreased after JMR-132 treatment, which means that the antiproliferative effect of JMR-132 might be associated with EGFR. It is well known that the activation of EGFR leads to the activation of PI3K, which in turn activates Akt, the main downstream target, which appears to play various important roles in regulating cellular growth and apoptosis [48]. Therefore, to characterize the cell growth inhibition due to the decrease in EGFR protein, we measured EGFR downstream signaling of the Akt pathway by Western blotting. In accord with the decrease in EGFR level, treatment with JMR-132 induced a down-regulation of phosphorylation of Akt. More importantly, JMR-132 could attenuate the EGF-induced, upregulated p-Akt level. It was also observed that, treatment of SKOV3 and CaOV3 cells with JMR-132 alone, as well as co-treatment with EGF or treatment with LY294002, a PI3K/Akt inhibitor, the pro-proliferative effect of EGF was all abolished by JMR-132. These results demonstrate that the down-regulation of the EGFR-Akt pathway which was induced by JMR-132 treatment results in inhibition of cell proliferation. In addition, it was reported that Akt can directly inhibit caspase9 and caspase3 to avoid apoptosis and cleaved caspase3 level was activated and increased by decreased p-Akt level [51]. Then we examined the effect of EGFR-Akt pathway in apoptotic process. Similar results were seen with cleaved caspase3 level. The expression of cleaved caspase3 was still increased after treatment with JMR-132 in combination with EGF. JMR-132 and EGF co-treatment were able to counter the EGF protection of cells, which led to an increase in cleaved caspase3, suggesting that apoptosis still occurred. The data demonstrate that down-regulation of the EGFR-Akt pathway induced by JMR-132 treatment is the main contributor in suppression of cell proliferation and induction of apoptosis.

These observations prompted us to investigate whether the antiproliferative effect of JMR-132 could be altered if endogenous EGFR was eliminated. The results confirmed our hypothesis as there was no difference between the siControl and siEGFR groups after treatment with JMR-132. The same findings were observed for cleaved caspase3 expression. These data suggest that the antiproliferative effect of JMR-132 is abolished if EGFR expression is knocked down, confirming that the antiproliferative effect of JMR-132 is due to the reduction in EGFR. Thus, our findings indicate that the effect of JMR-132 on proliferation may partially be mediated through the EGFR pathway.

Of much interest was the observation of slowed growth and cleaved caspase 3 induction in SKOV3 and CaOV3 cells after EGFR knock-down, which indicates that a decreased level of EGFR results in apoptosis and decreased proliferation. Furthermore, the effects of JMR-132 growth inhibition in CaOV3 cells were higher in the EGFR siRNA group when compared to the control siRNA group after JMR-132 treatment. All these results point to the critical role of EGFR in cell proliferation. Therefore, the inhibition of EGFR level by JMR-132 could be of significance in clinical treatment ovarian cancer.

## Conclusions

In summary, we show here for the first time that the GHRH antagonist JMR-132 acts as an effective anti-proliferation agent in the ovarian cancer cell lines, SKOV3 and CaOV3, by inducing apoptosis. Furthermore, we also shed light on the potential molecular mechanism of JMR-132, which may occur through partial suppression of the EGFR-Akt pathway.

## Competing interests

The authors declare that they have no competing interests.

## Authors' contributions

JG designed the study and performed the experiments and participated in discussion of the results and drafted the manuscript. AVS, MZ and JV provided the reagent of JMR-132, and were responsible for supervision of this work. PCKL was responsible for the conception, design, discussion of the results, drafting and critical revision of the manuscript. All authors read and approved the final manuscript.
